# Bacteriophage therapy against American foulbrood in honey bees: a PRISMA-guided systematic review

**DOI:** 10.3389/fmicb.2026.1816537

**Published:** 2026-06-01

**Authors:** Nike Walter, Aaron Teja Bolte, Mohammadali Khan Mirzaei, Li Deng, Markus Rupp

**Affiliations:** 1 Department for Trauma Surgery, University Hospital Regensburg, Regensburg, Germany; 2 VOROS Fund for Regenerative Innovation, Vaduz, Liechtenstein; 3 Institute of Virology, Helmholtz Centre Munich - German Research Centre for Environmental Health, Neuherberg, Germany; 4 TUM School of Life Sciences, Central Institute of Infection Prevention (ZIP), Technical University of Munich, Freising, Germany; 5 Department of Trauma, Hand and Reconstructive Surgery, University Hospital Giessen, Giessen, Germany

**Keywords:** American foulbrood, antimicrobial resistance, bacteriophage, honey bee, *Paenibacillus larvae*

## Abstract

American foulbrood (AFB), caused by the spore-forming bacterium *Paenibacillus larvae*, is the most destructive bacterial brood disease of *Apis mellifera*. Control is challenging because endospores persist for decades and antibiotic use is restricted by regulations, resistance, and residue concerns. Bacteriophages have re-emerged as a species-specific, residue-free strategy for AFB prevention and treatment. A PRISMA-guided systematic review was conducted searching PubMed, Embase, and Web of Science using the terms (bacteriophage OR phage) AND (honeybee OR *Apis mellifera*). A total of 90 records were retrieved; after deduplication (19 removed), 71 titles and abstracts were screened. Eleven full texts were assessed for eligibility. Nine studies met inclusion criteria: three in adult bees or colonies, four in larvae, and two *in vitro*. Across studies, 27 *P. larvae* phages were characterized *in vitro*, 18 tested in larval assays, and nine evaluated at the hive level, mainly via oral delivery. In a controlled hive trial, a three-phage cocktail provided complete protection from natural AFB (100% protected vs. 80% infection in controls; *p* < 0.05) and enabled full recovery within 2 weeks, performing comparably to or better than prophylactic antibiotics. Larval studies generally showed significantly improved survival with prophylactic or therapeutic dosing, though efficacy varied by phage. A biodistribution study found limited indirect larval exposure after adult feeding (~32 PFU/larva at 24 h), indicating hive-mediated inactivation as a key translational barrier. One phage bound both vegetative cells and spores while retaining ~93% *in-vitro* infectivity. Overall, phage cocktails show strong proof-of-concept, with formulation, delivery, and rational cocktail design as key translational priorities.

## Graphical abstract

**Figure:**
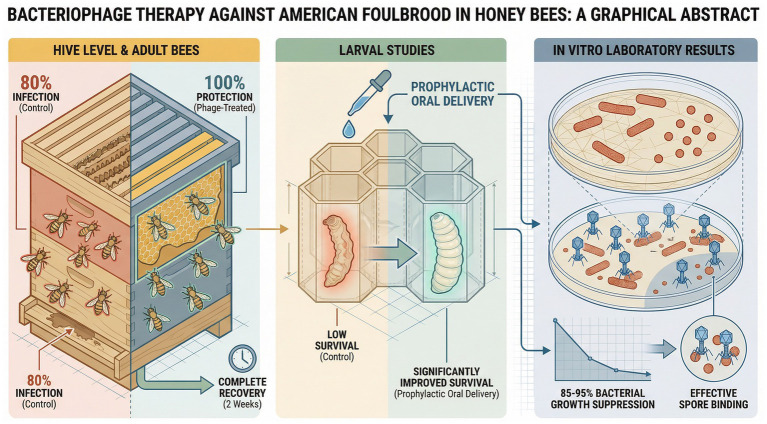

## Introduction

Honey bees (*Apis mellifera*) are indispensable to global food security and ecosystem stability through their central role in pollination (Hung et al., 2018). However, sustained declines in managed honey bee populations have been reported worldwide, driven by the synergistic effects of environmental stressors, parasites, and infectious diseases. Among these, American foulbrood (AFB) remains the most destructive bacterial disease of honey bee brood, posing a persistent threat to apiculture and agricultural productivity (Mill et al., 2014). AFB is caused by the Gram-positive, spore-forming bacterium *Paenibacillus larvae*, whose extraordinary environmental resilience and high infectivity render disease control exceptionally challenging (Zabrodski et al., 2022).

*P. larva* produces highly durable endospores that can remain viable for decades in hive materials and honey, resist heat and chemical disinfectants, and rapidly disseminate within and between colonies. Conventional antimicrobial strategies are fundamentally limited, as antibiotics target only vegetative bacterial cells and fail to eliminate spores (Rodrigues et al., 2024). Moreover, the emergence of antibiotic-resistant *P. larvae* strains and the accumulation of antibiotic residues in honey products have led to strict regulatory bans on antibiotic use in apiculture across much of Europe and other regions (Matović et al., 2023). Consequently, the destruction of infected colonies remains the primary control strategy in many countries, resulting in substantial economic and ecological losses (Agriculture and Forestry, Government of Alberta, 2020). Against this backdrop, bacteriophages have re-emerged as a compelling alternative for the control of AFB. Phages are highly specific bacterial viruses that self-amplify at the site of infection and exert minimal impact on non-target microbiota.

A landmark review by Jończyk-Matysiak et al. (2020) comprehensively summarized early knowledge on phage therapy and prophylaxis for AFB, focusing on phage diversity, basic biological properties, and first-generation application attempts (Jończyk-Matysiak et al., 2020). The present review extends this foundation in several critical respects: it applies a formal PRISMA 2020 framework for systematic literature synthesis; it incorporates studies published after 2020, including controlled larval infection experiments, full colony-level hive trials, phage stability and formulation analyses, and the first investigation of phage resistance dynamics in *P. larvae* (Spencer et al., 2025); and it explicitly evaluates practical translational barriers—delivery constraints, hive-environment inactivation, and rational cocktail design—that prior reviews did not address in depth. Furthermore, the present review situates *P. larvae* phage research within the broader phage therapy literature by drawing on cross-disciplinary evidence from preclinical and clinical phage therapy studies, identifying transferable principles for formulation and resistance management.

The aim of this review is therefore to systematically evaluate bacteriophage-based strategies for the control of American foulbrood in honey bees, with an emphasis on experimental efficacy, delivery routes, phage stability, and resistance development across *in vitro*, larval, and colony-level studies. By integrating findings across biological scales, this review seeks to identify general principles governing phage performance in complex biological systems and to highlight factors that constrain or enable successful phage application.

## Methods

This review was conducted as an updated systematic review following the principles of the Preferred Reporting Items for Systematic Reviews and Meta-Analyses (PRISMA) 2020 guidelines.

### Search strategy and selection criteria

A comprehensive literature search was performed in three major bibliographic databases: PubMed, Embase and Web of Science (WoS). These databases were selected to ensure broad coverage of microbiology, entomology, veterinary science, and applied phage therapy research. The final literature search was conducted on 01.02.26. The search strategy was designed to identify studies addressing bacteriophages in honey bees and was applied using the following Boolean logic: (bacteriophage OR phage) AND (honeybee OR *Apis mellifera*). The exact search strings applied in each database were as follows. In PubMed: (bacteriophage[tiab] OR phage[tiab]) AND (honeybee[tiab] OR “honey bee”[tiab] OR “*Apis mellifera*”[tiab] OR “*Paenibacillus larvae*”[tiab] OR “American foulbrood”[tiab] OR AFB[tiab]). In Embase: (‘bacteriophage’:ab,ti OR ‘phage’:ab,ti) AND (‘honeybee’:ab,ti OR ‘honey bee’:ab,ti OR ‘*Apis mellifera*’:ab,ti OR ‘*Paenibacillus larvae*’:ab,ti OR ‘American foulbrood’:ab,ti). In WoS: TS = ((bacteriophage OR phage) AND (honeybee OR “honey bee” OR “*Apis mellifera*” OR “*Paenibacillus larvae*” OR “American foulbrood” OR AFB)). Searches were conducted without restrictions on publication year to capture both foundational and recent studies. Only articles published in English were considered. The final search retrieved 90 records prior to deduplication. All identified records were imported into reference management software, and duplicate entries were removed prior to screening (*n* = 19). The remaining 71 records were screened based on titles and abstracts to assess relevance to bacteriophage applications in honey bees, honey bee larvae, American foulbrood, or *Paenibacillus larvae*. Title and abstract screening was performed independently by two reviewers (NW and ATB). Disagreements were resolved by discussion and, if necessary, consultation of a third reviewer (MR). Full-text eligibility assessment followed the same dual-reviewer process. Studies on phage-derived products (e.g., endolysins) were excluded for the scope of this article. During the screening stage, 60 records were excluded because they did not meet the inclusion criteria, primarily due to irrelevance to phage-based approaches, focus on unrelated pathogens, or absence of application to honey bees. Full-text articles were sought for the 11 remaining records. All reports were successfully retrieved and assessed for eligibility. Two studies were excluded at this stage because only an abstract was available and no full-text article could be accessed (Poster Section 14: Immunopharmacology and Biologics, 2018). Studies were included in the review if they met the following criteria: (1) Investigated bacteriophages, phage therapy addressing *P. larvae*, American foulbrood, or honey bee (*Apis mellifera*) health, (2) Presented original experimental data (*in vitro*, *in vivo*, or hive-level), genomic analyses, or applied/prophylactic evaluations, (3) were available as full-text, one preprint article included (Spencer et al., 2025).

### Data extraction

The following data fields were extracted from each included study: first author and year, country of origin, study design (*in vitro*/larval/colony-level), phage(s) tested (names and family), bacterial strains or isolates tested (*n*), phage preparation and delivery method, titer (PFU/mL), stability data if reported, and primary outcome(s).

### Data analysis

A meta-analysis was not performed due to significant heterogeneity in study designs, infection models, and outcome reporting; therefore, results were synthesized narratively.

### Quality assessment

In the absence of a meta-analysis, study quality was assessed descriptively using the following criteria: adequacy of controls, sample size, blinding, and reproducibility of the phage preparation. No formal risk-of-bias tool was applied given the heterogeneity of study types.

## Results

**Figure 1:**
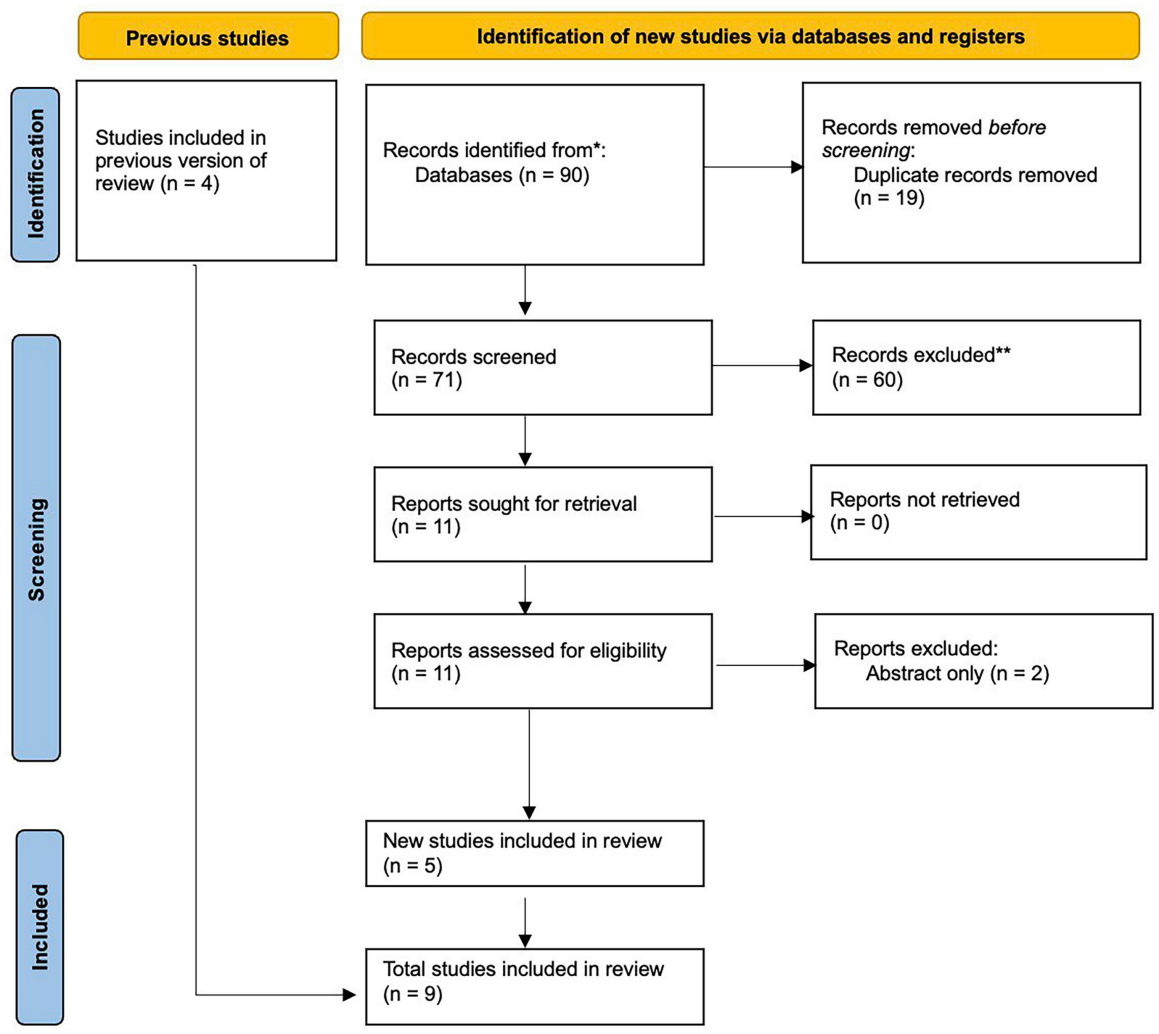
PRISMA flow diagram for updated systematic reviews (Page et al., 2021), showing records retrieved (*n* = 90), deduplicated (*n* = 71), full texts assessed (*n* = 11), and included studies (*n* = 9).

Nine studies met the predefined inclusion criteria and were included in the final synthesis (Figure 1). These comprised three *in vivo* studies in adult honey bees, four *in vivo* studies in honey bee larvae, and two *in vitro* studies focusing on bacteriophage characterization or efficacy against *Paenibacillus larvae*. Across all included studies, a total of 27 distinct *P. larvae* bacteriophages were characterized *in vitro*, 18 were applied in larvae experiments, and 9 in hive-level contexts.

Three studies evaluated bacteriophage administration in adult honey bees, either to assess safety, biodistribution, or colony-level efficacy (Table 1). Jończyk-Matysiak et al. (2021) administered individual phages and a two-phage cocktail orally in sucrose solution to adult bees. Phages belonged to the *Siphoviridae* family and were tested against 35 *P. larvae* isolates. Phage preparations at approximately 10^5^ PFU/mL (10% sucrose) and 10^3^ PFU/mL (1% sucrose) showed prolonged stability at 4 °C for up to 250 days, with lyophilized formulations remaining viable for 125 days, except for one phage. The authors also determined the purification level (EU/mL) for all phages. No adverse effects on adult bee survival were observed, and bees receiving the phage cocktail exhibited significantly lower mortality compared with bees receiving only one phage, as well as compared to controls (Jończyk-Matysiak et al., 2021). Ribeiro et al. (2019) investigated phage biodistribution following oral administration of a T7 phage model in six colonies. While phages were readily detected in adult bee tissues after feeding, only a limited number reached larvae indirectly via nurse bees, with an average of 32 PFU per larva detected after 24 h. These findings highlighted substantial phage inactivation within the hive environment, particularly in royal jelly, suggesting a potential limitation of indirect larval delivery strategies. It is indicating that while oral delivery is feasible, phage protection or stabilization strategies are required for effective AFB control (Ribeiro et al., 2019). At the colony scale, Brady et al. (2017) evaluated a three-phage cocktail administered orally in sugar syrup. Phage-treated hives showed outcomes comparable to or better than prophylactic antibiotic treatment, with no significant negative effects on overall hive health. The cocktail was considered safe for honeybees, even at overdose levels. Notably, 100% of phage-treated hives were protected from natural AFB infection, whereas 80% of untreated controls became infected (*p* < 0.05). In infected colonies, complete recovery was observed within 2 weeks of phage treatment (Brady et al., 2017).

**Table 1:** *In vivo* studies in adult bee.

Author, Year	Origin	Cohort (*n*)	Phages used	Phage family	Prepared in	Isolates tested (*n*)	Titer (PFU/mL)	Stability	Outcome
Jończyk-Matysiak et al. (2021)	Poland	*n* = 30/group adult bee	1/A (P1), 2/A, 3/A (P2), 4/A, 5/A 1/A + 3/A (P3)	*Siphoviridae*	50% sucrose, orally fed	35	10% preparation ~10^5^ 1%, ~10^3^	4°C, 250 days + Lyophilized 125 days 4/A not stable	No negative effects, lowest mortality in P3 cocktail group, *p* < 0.05
Ribeiro et al. (2019)	Portugal	6 colonies/*n* = 30 adult bee	T7	*Autographiviridae*	50% (w/v) sucrose, orally fed	–	10^9^	24 h	Phages are uptake by adult bees, quantity of phages per larvae was only 32 PFU/larvae
Brady et al. (2017)	USA	96 hive *n* = 32/group	1, 5, 9 cocktail	*Siphoviridae*	sugar syrup, orally fed	59	10^6^	–	Comparable bee health prophylactic antibiotics vs. vs. control (n.s) 100% protected vs. 80% of control became infected, *p* < 0.05; 100% of the hives recovered within 2 weeks

Four studies directly assessed phage efficacy in honey bee larvae under controlled laboratory conditions (Table 2). All studies involved oral delivery of phages mixed into larval diet. Yost et al. (2016) tested seven *Siphoviridae* phages against 11 *P. larvae* isolates and demonstrated significantly improved larval survival following phage treatment compared with infected controls (*p* < 0.05; Yost et al., 2016). Similarly, Ghorbani-Nezami et al. (2015) reported that larvae treated with phages F and WA exhibited survival rates comparable to uninfected controls, with phage administration proving more effective as a prophylactic than as a post-infection treatment (Ghorbani-Nezami et al., 2015). Beims et al. (2015) evaluated the phage HB10c2, which showed strong lytic activity *in vitro* but failed to provide consistent protection in larval infection assays. This discrepancy underscored the limited predictive value of *in vitro* lytic activity alone for therapeutic efficacy (Beims et al., 2015). The most recent study by Spencer et al. (2025), published as a preprint, investigated the evolutionary dynamics of phage resistance using seven closely related phages applied to larvae infected with *P. larvae*. While phage-resistant bacterial variants emerged, the isolate exhibited reduced pathogenicity in larvae and showed growth defects, suggesting a fitness trade-off associated with resistance. The authors further reported that resistance to one phage did not confer broad cross-resistance, supporting the rationale for phage cocktail approaches. In addition, some phages retained the ability to overcome resistance, indicating that ongoing phage evolution could help mitigate resistance problems (Spencer et al., 2025).

**Table 2:** *In vivo* studies in ***P. larvae***.

Author, Year	Origin	Phages used	Phage family	Prepared in	Isolas tes pos. tested	Outcome
Spencer et al. (2025)	USA	vB_Pla3650_Fern_IDv1, vB_Pla3650_Xeni_IDv1, vB_Pla3650_Xeni_IDv2, vB_Pla3650_Will_IDv1, vB_Pla3650_Heat_IDv1, vB_Pla3650_Scot_IDv1, vB_Pla3650_Unit_IDv1	-	mBHI, orally fed	1 phage-susceptible 1 phage-resistant	reduced pathogenicity in the resistant variant, suggested beneficial trade-off
Yost et al. (2016)	USA	Hayley, Xenia, Fern, Willow, Halcyone, Vadim, Harrison	*Siphoviridae*	GmBHI, orally fed	11	improved survival, *p* < 0.05
Ghorbani-Nezami et al. (2015)	USA	F, WA XIII	*Siphoviridae*	Buffered aqueous solution, orally fed	1	improved survival, *p* < 0.05
Beims et al. (2015)	Germany	HB10c2	*Siphoviridae*	Mixed directly in diet, orally fed	4	HB10c2 is lytic *in vitro* but did not show consistent therapeutic protection

Two studies focused primarily on *in vitro* phage discovery, host range determination, and efficacy testing (Table 3). Kok et al. (2023) isolated 26 novel *P. larvae* phages in New Zealand using a community science framework. Host range testing across eight bacterial isolates informed the design of prophylactic phage cocktails, which reduced bacterial growth by approximately 85–95% *in vitro.* Genomic analyses confirmed substantial diversity among isolated phages, reinforcing their suitability for cocktail formulation (Kok et al., 2023). Brady et al. (2021) characterized the phage PL.Ph-1 and demonstrated efficient binding to vegetative *P. larvae* cells and strong binding to dormant spores, despite spores being metabolically inactive, using flow cytometry and electron microscopy. Phage binding to spores was reversible, and phages retained infectivity upon subsequent exposure to vegetative cells, achieving approximately 93% efficacy *in vitro*. These findings provided a mechanistic explanation for the prolonged protection observed in phage-treated hives (Brady et al., 2021).

**Table 3:** *In vitro* studies.

Author, Year	Origin	Phages	Strains tested	Outcome	Efficacy
Kok et al. (2023)	New Zealand	ABAtENZ AJG77 ApiWellbeing BarryFoster_Benicio Bloomfield Bob Callan Carlos Dante Dash FutureBee GaryLarson GIW2016 Jacinda Lena Lilo Logan LunBun NHScienceFair Ollie Rae.2Bee1 Rosalind Ted TonyLawson77 UtuhinaGold_Zacery WildCape	8	Feasibility of prophylactic phage cocktails	≈85–95% growth inhibition vs. untreated controls
Brady et al. (2021)	USA	PL.Ph-1	1	PL.Ph-1 binds efficiently to vegetative *P. larvae* cells by flow cytometry, remains fully infective	~93%

### Thematic synthesis

Delivery routes: Across all nine included studies, oral delivery via sucrose solution or sugar syrup was the universal administration route for both adult bees and larvae. No study evaluated direct brood comb application or encapsulated formulations, identifying this as a gap.

Efficacy across biological scales: *In vitro* efficacy (85–95% growth inhibition, Brady et al., 2021: ~93% infectivity retention) did not consistently translate to larval protection—Beims et al. (2015) demonstrated this gap directly. Colony-level efficacy was demonstrated in one controlled trial (Brady et al., 2017), with 100% protection in phage-treated hives.

Stability: Only one study systematically assessed phage stability (Jończyk-Matysiak et al., 2021), reporting viability at 4 °C for up to 250 days in liquid and 125 days lyophilized. Stability data under field-relevant temperatures are absent across all other studies.

Resistance dynamics: Spencer et al. (2025) provided the only direct examination of resistance evolution, demonstrating emergence of resistant *P. larvae* variants accompanied by reduced pathogenicity—a fitness trade-off that may limit the clinical relevance of resistance in this system.

## Discussion

This systematic review indicates that bacteriophage-based interventions against AFB have advanced from concept to practical viability. The nine included studies, spanning *in vitro* assays, larval infection models, and full hive trials, collectively demonstrate that phages can significantly reduce *P. larvae* infection and improve honey bee survival. Phage treatments protected larvae from lethal AFB in controlled experiments and even prevented disease outbreaks in colonies, matching or exceeding the efficacy of traditional antibiotic prophylaxis. Notably, no adverse effects on bee health or colony performance were reported; phages were well-tolerated by adult bees and brood in all trials. Bacteriophages thus emerge as a highly promising and environmentally benign tool for AFB management.

Phage therapy addresses several critical shortcomings of conventional antimicrobial strategies in apiculture. Antibiotics such as oxytetracycline have long been employed to suppress clinical AFB symptoms; however, they fail to eliminate the highly resilient spore form of *P. larvae* and therefore often merely delay disease recurrence rather than achieving eradication (Jończyk-Matysiak et al., 2020). Moreover, decades of prophylactic antibiotic use have driven the emergence of resistant *P. larvae* populations; for instance, approximately 9% of isolates in a recent Canadian survey exhibited high-level resistance to oxytetracycline (Obshta et al., 2023). Despite the regulatory prohibition of antibiotic use in European apiculture, a recent review has identified a pooled concentration of antibiotic residues in honey of up to 5.33 μg/kg. These consisted of fluoroquinolone (8.59 μg/kg), tetracycline (5.68 μg/kg), sulfonamides (5.54 μg/kg), and macrolides (4.19 μg/kg; Shoaei et al., 2024). In stark contrast, bacteriophages provide a highly targeted antimicrobial approach that leaves no chemical residues in hive products and preserves non-target microbial communities, thereby aligning closely with contemporary food safety and regulatory expectations. Critically, these conceptual advantages of phage therapy are supported by direct comparative efficacy data. Brady et al. (2017) provided the first controlled hive-level evidence demonstrating that a three-phage cocktail conferred protection against AFB that was equivalent to, or exceeded, that achieved by prophylactic antibiotic treatment (Brady et al., 2017). This finding establishes practical efficacy parity with the historical pharmaceutical standard in regions where antibiotic use remains permissible, while simultaneously avoiding the regulatory, ecological, and resistance-associated liabilities of antibiotics.

### Translational aspects

Looking ahead, several steps remain to translate these promising findings into a field-ready AFB phage therapy product. Stability of phage preparations under typical hive temperatures and conditions is another key consideration – encouragingly, one study showed that phage suspensions in a sucrose solution remained viable for months when stored cold (Jończyk-Matysiak et al., 2021). Developing formulations (possibly lyophilized powders or slow-release carriers; Huang et al., 2025) that beekeepers can easily apply may enhance uptake of the technology. Another issue is delivering phages effectively to the brood. When phages are simply fed to adult bees, only a scant fraction (on the order of tens of PFU) reaches the larvae via nurse bee secretions (Ribeiro et al., 2019). Many phage particles are inactivated by the harsh hive environment—notably by components of royal jelly—before they can impact the brood infection. This highlights the need for improved delivery methods or formulations (e.g., micro-encapsulation, controlled-release feeding, or direct application to brood comb) to protect phages until they contact larval gut targets (Vila et al., 2024).

Another challenge is that not every lytic phage that looks promising *in vitro* will prove effective *in vivo*. Beims et al. (2015) found that a highly active phage (HB10c2) with broad *in vitro* lytic capacity failed to significantly improve survival of infected larvae in laboratory assays (Beims et al., 2015). The lack of therapeutic effect in that case underscores the importance of screening phage candidates under realistic conditions. This disconnect between *in vitro* lytic activity and *in vivo* protection is not unique to this system; it represents a fundamental limitation of reductionist screening approaches that do not account for phage stability in complex biological matrices, physical barriers to diffusion, or immunological clearance. As such, *in vitro* lysis profiles should be considered a necessary but insufficient criterion for candidate selection in translational phage therapy programs, whether for apicultural or clinical applications.

The potential for *P. larvae* to evolve phage resistance is another consideration. Laboratory evolution studies confirm that *P. larvae* can develop resistance mutations to infecting phages relatively quickly, but often at a significant cost to bacterial fitness (Spencer et al., 2025). In essence, the trade-off for the bacterium to become phage-resistant appeared to be a loss of pathogenicity. Moreover, bacteriophages are not static agents—they can mutate and evolve alongside their host. This co-evolutionary dynamic, combined with the use of multi-phage cocktails that broaden the host range, means phage therapy is inherently adaptable. Over time, a well-designed phage cocktail can be updated to counter new *P. larvae* strains if needed, a flexibility that conventional antibiotics lack. In practical terms, while phage resistance may arise, its impact is likely to be mitigated by reduced bacterial virulence and the capacity to introduce fresh phages—a fundamentally different scenario than the one-dimensional rise of antibiotic resistance. The findings of this review also support rational cocktail design: the observation by Kok et al. (2023) that phylogenetically diverse phages achieved 85–95% growth suppression *in vitro*, combined with Brady et al. (2017)’s field success with a three-phage cocktail, establishes proof-of-concept for multi-component formulations (Brady et al., 2017; Kok et al., 2023). Continued phage discovery and library expansion may ensure that phage cocktails can be tailored to regional *P. larvae* strains.

### Regulatory pathway

In most jurisdictions, phage-based products for veterinary/apicultural use fall into an undefined regulatory category—neither classical veterinary medicinal product nor conventional pesticide. In the European Union, phage products for animal use would likely require authorization under Regulation (EU) 2019/6 on veterinary medicinal products, necessitating documented quality, safety, and efficacy data. In the United States, phage-based treatments for honeybee disease might be regulated by the USDA or EPA depending on the product’s classification. Proactive pre-submission dialogue with regulators and the development of standardized quality criteria (phage identity, titer, purity, stability) will be essential to advance any candidate product toward commercialization.

### Manufacturing and quality considerations

Reproducible, scalable phage production under GMP-equivalent conditions presents a significant challenge for biologics of this type. Key parameters include consistent phage titering, endotoxin removal (particularly relevant for food-product-adjacent applications), long-term storage stability, and batch-to-batch reproducibility. The studies reviewed here used laboratory-scale preparations with variable purity assessment; only Jończyk-Matysiak et al. (2021) reported endotoxin levels (EU/mL) for their phage preparations, which should be considered a minimum quality benchmark.

### Field deployment

From a beekeeper perspective, practical uptake will depend on ease of application, cost, availability, and clarity of dosing guidance. Formulations that can be mixed into standard sugar syrup feeders with defined dosing schedules (e.g., prophylactic feeding at the start of the foraging season) would minimize behavioral change requirements. Engagement of beekeeping associations in field trial design and product testing—as demonstrated by the community-science approach of Kok et al. (2023)—represents a promising model for ensuring that phage products are both scientifically validated and practically acceptable.

### Implications for phage therapy in human medicine

The findings summarized in this review extend beyond apiculture and provide mechanistically grounded insights that offer analogies relevant to current challenges in human phage therapy, particularly with respect to delivery, resistance evolution, and cocktail design. A central parallel concerns delivery barriers: the rapid inactivation of *Paenibacillus larvae* phages during biological transfer within the hive—most notably by components of royal jelly, resulting in only minimal phage numbers reaching larvae after adult feeding—mirrors a critical limitation observed in human applications. In humans, orally or systemically administered phages are frequently degraded by digestive enzymes, sequestered by mucosal barriers, or cleared by innate immune mechanisms, such that therapeutically relevant concentrations often fail to reach infection sites in the gastrointestinal tract, chronic wounds, or anatomically compartmentalized infections such as bone or joint biofilms (Wang et al., 2025). Together, these observations underscore that phage efficacy is constrained less by intrinsic lytic capacity than by successful delivery through hostile biological environments, identifying formulation and controlled release as primary translational determinants.

Importantly, recent advances in human phage therapy demonstrate that these barriers can be overcome through formulation-based solutions. Encapsulation strategies, including pH-responsive hydrogels, liposomes, nanoparticles and biomaterial-assisted delivery systems, have been shown to preserve phage viability under enzymatically harsh conditions, prolong systemic half-life, and improve tissue penetration, resulting in markedly enhanced therapeutic outcomes compared with free phage administration (Yue et al., 2025). These clinical findings support the transferable principle that phage therapy success depends on formulation-mediated protection and spatiotemporal control rather than phage potency alone. Similarly, the discrepancy observed between strong *in vitro* lytic activity and limited *in vivo* protection in larval infection assays highlights a universal limitation of phage screening strategies. This limitation, observed in the honey bee system, is analogous to findings in clinical phage research, where in vitro lysis profiles have similarly been found to be insufficient predictors of therapeutic utility unless complemented by biologically relevant models that capture environmental inactivation, host interactions, and spatial constraints (Dorta-Gorrín et al., 2026).

The review further emphasizes the translational importance of multi-phage cocktail design. The superior performance of phage cocktails at the colony level, together with *in vitro* evidence of enhanced growth suppression by phylogenetically diverse phage combinations, parallels accumulating clinical evidence that rationally designed cocktails outperform single-phage approaches in heterogeneous and evolving infection landscapes. Cocktails broaden host range, buffer against resistance emergence, and provide functional redundancy when individual phages fail (Kaneko et al., 2026). This principle is broadly supported by systematic evidence from non-apicultural systems: a meta-analysis of phage therapy in preclinical bacterial infection models reported significant efficacy of phage interventions, while simultaneously identifying high inter-study heterogeneity, variable dosing strategies, and inconsistent outcome reporting as the primary barriers to generalization (Gómez-Ochoa et al., 2022). Similarly, a recent meta-analysis found that phage therapy outperformed controls across a range of bacterial pathogens, reinforcing the residue-free advantage of phage approaches as an antibiotic alternative (Aloufi, 2025). While these meta-analyses focus on non-apicultural systems, the barriers they identify—heterogeneous study designs, delivery challenges, and resistance dynamics—are strikingly paralleled in the present honey bee review, suggesting shared principles worth exploring across systems.

Notably, the adaptability of phage libraries—exemplified by iterative discovery and selection efforts in the honey bee system—mirrors emerging clinical paradigms in which phage formulations are dynamically updated in response to pathogen evolution, a flexibility fundamentally absent from conventional antibiotic development (Gibson et al., 2019). The evolution of phage resistance in *P. larvae*, frequently accompanied by reduced pathogenicity or fitness costs, supports a broader paradigm shift in how resistance is interpreted in phage therapy. Rather than representing inevitable therapeutic failure, resistance may alter host–pathogen dynamics in ways that reduce virulence or restore susceptibility to other control measures (Chen et al., 2024). This concept is increasingly substantiated in human infections, where phage-resistant variants often exhibit impaired fitness, altered surface structures, or renewed antibiotic sensitivity (Zhang and Ahn, 2025).

### Limitations

Despite increasing interest in bacteriophage-based interventions against American foulbrood, several limitations emerge from the current body of evidence that constrain both interpretation of existing results and translational potential. Most studies to date rely on laboratory or semi-controlled experimental systems. While these models are essential for proof-of-concept and mechanistic understanding, they do not fully capture the complexity of natural hive environments. Factors such as colony dynamics, seasonal variation, microbiome interactions, and beekeeper management practices are rarely addressed. Consequently, the real-world efficacy and durability of phage-based interventions at the apiary level remain insufficiently characterized. Thus, larger field trials under diverse climatic and management conditions will be crucial to validate efficacy at commercial apiary scales. Such trials should also address practical questions like optimal dosing frequency (e.g., monthly prophylactic feeding vs. only upon disease detection) and application methods (feeding phage-laced syrup, spraying brood frames, etc.). Only three studies directly assessed phage efficacy at the colony level, limiting statistical power and generalizability of hive-based findings. The mechanistic basis for heterogeneous efficacy among different phages (e.g., why PL.Ph-1 shows exceptional host range) remains incompletely understood. Further, long-term population dynamics of phage-treated colonies over multiple seasons have not been systematically evaluated; colonies protected from infection in year 1 might face different infection pressure in year 2 if phage-resistant variants accumulate, or conversely, might experience sustained protection through continued phage replication.

## Conclusion

The current evidence supports phage cocktails as a promising, residue-free, and potentially antibiotic-comparable strategy for AFB control, with strong proof-of-concept demonstrated at the colony scale. Translating this promise into routine practice will require coordinated progress across several dimensions.

From a formulation standpoint, the development of standardized phage cocktails with well-characterized host ranges and reproducible manufacturing processes is a priority. Cocktail composition should be based on phylogenetically diverse phage combinations shown to minimize cross-resistance and maximize coverage of regional *P. larvae* strains. Stability studies under realistic hive conditions—including temperature fluctuations, UV exposure, and interaction with hive products such as royal jelly and propolis—are essential to define shelf life and application windows for field use.

At the applied level, phage-based interventions should be designed for integration with existing colony management practices, including routine hive inspections, biosecurity protocols, and disease monitoring. Practical delivery formats—such as lyophilized powders mixed into sugar syrup or slow-release feeding inserts—would lower the barrier to beekeeper adoption. Regulatory pathways for phage-based veterinary products in apiculture remain undefined in most jurisdictions; proactive engagement with regulatory agencies (e.g., the EMA in Europe, the USDA/EPA in North America) will be necessary to define approval requirements for phage cocktails as biological control agents.

Finally, longitudinal field studies spanning multiple seasons are critically needed to evaluate phage persistence in hive environments, monitor the emergence and fitness cost of phage-resistant *P. larvae* variants, and assess long-term colony health outcomes. Until such evidence is available, phage therapy must be recognized as a highly promising, residue-free alternative to antibiotics, but one that requires rigorous field validation before routine adoption in commercial apiculture.
